# Seasonal Variations of the Nutritive Value and Phytotherapeutic Potential of *Cladium mariscus* L. (Pohl.) Targeting Ruminant’s Production

**DOI:** 10.3390/plants10030556

**Published:** 2021-03-16

**Authors:** Marta Oliveira, Maria João Rodrigues, Nuno R. Neng, José Manuel Florêncio Nogueira, Rui J. B. Bessa, Luísa Custódio

**Affiliations:** 1 Centre of Marine Sciences, Universidade do Algarve, Campus de Gambelas, 8005-139 Faro, Portugal; mmfoliveira11@gmail.com (M.O.); mary_p@sapo.pt (M.J.R.); 2 Centro de Química Estrutural, Faculdade de Ciências, Universidade de Lisboa, Campo Grande, 1749-016 Lisboa, Portugal; ndneng@fc.ul.pt (N.R.N.); jmnogueira@fc.ul.pt (J.M.F.N.); 3 CIISA—Centro de Investigação Interdisciplinar em Sanidade Animal, Faculdade de Medicina Veterinária, Universidade de Lisboa, Avenida da Universidade Técnica, 1300-666 Lisboa, Portugal; rjbbessa@fmv.ulisboa.pt

**Keywords:** salt-tolerant plants, nutritional profile, phenolics, antioxidant, anti-inflammatory, veterinary

## Abstract

In our endeavor to identify salt-tolerant plants with potential veterinary uses in ruminants’ production strategies, we focused on *Cladium mariscus* L. Pohl (sawgrass), due to its high total phenolic and tannin content, anti-radical properties, and ethnomedicinal uses. Aerial parts were collected along the year in Southern Portugal and evaluated for the nutritional profile and in vitro organic matter digestibility (IVOMD), aiming for its use as feed. Acetone extracts were appraised for total contents in phenolics (TPC), flavonoids (TFC), and tannins (CTC), as well as the chemical composition by HPLC-DAD and in vitro antioxidant and anti-inflammatory properties, targeting its exploitation as phytotherapeutic products. Sawgrass biomass has a limited nutritive value, due to its high neutral detergent fiber (NDF; 596–690 g kg^−1^ dry matter (DM)) and acid detergent fiber (ADF; 330–418 g kg^−1^ DM) contents, low crude protein (51.8–87.3 g kg^−1^ DM) and IVOMD (172–317 g kg^−1^ organic matter (OM)). Despite differences among seasons, the mineral profile was adequate. The extracts were rich in TPC (88–112 mg g^−1^), CTC (115–169 mg g^−1^), and TFC (18.5–20.2 mg g^−1^), and displayed significant antioxidant capacity, particularly in summer and autumn, whilst no seasonal influence was detected for anti-inflammatory properties (30% reduction of nitric oxide production). Eleven phenolics were quantified: chlorogenic, ferulic, and syringic acids were the most abundant, especially in the autumn sample. Overall, despite the low nutritional interest, sawgrass extracts hold the potential as a source of antioxidant and anti-inflammatory phenolic compounds.

## 1. Introduction

Climate changes will have a strong impact on the Mediterranean area by significantly increasing drought, temperature, and evapotranspiration by the end of the 21st century [1,2]. This will negatively affect the agriculture and animal production by leading to soil and water salinization and degradation, freshwater scarcity, reduced crops yield and quality, and livestock losses [3].

Ruminants have a significant role in the Mediterranean Basin, and their production depends on costly feed supplementation strategies, mostly due to feed shortages during dry seasons [4]. The use of biomass from plants well adapted to that area’s constraints is considered an important strategy to reduce the costs associated with feed supplementation [5,6]. Moreover, there is an increasing effort to reduce the use of synthetic substances in livestock production, as supported by the European Parliament and Council (Regulation (EC) no. 1831/2003; Regulation (EC) no. 834/2007). This drives the interest on searching for bioactive plants and its products, such as salt-tolerant plants, as alternatives for the improvement of animal nutrition, health, and quality of its derived food products.

Salt-tolerant plants are adapted to several abiotic stresses, including high salinity and UV intensity and drought, in part due to the synthesis and accumulation of bioactive primary and secondary molecules with an important nutritive value and relevant biological properties, including antioxidant and anti-parasitic [7]. Some species are already used as feed resources in arid and semi-arid regions of the Mediterranean basin, for grazing animals, especially under drought conditions or to couple with seasonal pasture scarcity (e.g., *Sporobolus* sp.) [5]. Other species have ethnoveterinary uses [8], for example, *Pistacia lentiscus* L., as antiparasitic and for the treatment of bloat, constipation, and dermatological ailments. These plants are also characterized by high total levels of phenolic compounds, including tannins and flavonoids [9]. This latter aspect is particularly relevant, since plants with a high content of such compounds can, depending on the dose, exert multiple beneficial effects on animal health and performance, including anti-inflammatory, antioxidant, and anthelmintic effects, reduction of methane production, modulation of ruminal biohydrogenation, and improvement of the fatty acid content of meat products [6,10,11,12,13,14,15]. Therefore, salt-tolerant plants hold a high potential to be explored to develop novel nutritional and health management strategies for animal farming systems, especially in the context of climate change and soil and water salinization.

*Cladium mariscus* (L.) Pohl (Cyperaceae, sawgrass) is a perennial evergreen plant occurring in inland areas and coastal saltmarshes in the Mediterranean area and North Africa [16]. In ethnomedicine, it has been used to treat colds, renal pain, and colic in the gastrointestinal tract [17,18]. A previous screening of 21 extremophile plants from Southern Portugal identified *C. mariscus* leaves as a polyphenol-rich species and with strong anti-radical properties [9]. Nevertheless, to the best of our knowledge, the chemical assets and potential biological effects of sawgrass, as well as its seasonal variations were not priorly investigated. In this work, we hypothesized that this species could present interest for use as animal feed and/or to provide bioactive veterinary products to be included as part of production strategies to improve ruminants’ overall health. Thus, we conducted a nutritional evaluation to ruminants of *C. mariscus* biomass collected along the year coupled with a chemical and biological characterization of its extracts.

## 2. Material and Methods

### 2.1. Plant Collection and Processing

Sawgrass aerial parts (voucher code no. XBH03), including leaves and inflorescences, were manually harvested in Ludo, Faro, Southern Portugal (37°01′03.3″ N, 7° 59′18.1″ W) in spring (April 2017), summer (July 2017), autumn (October 2017), and winter (January 2018). Inflorescences were noted during summer and, to a lesser extent, in autumn, while green leaves were present all year. After collection, the samples were taken to the laboratory, washed, frozen at −20 °C, freeze-dried using a lyophilizer (Lyoalfa 15), and grinded using a ball miller (Retsch PM 100).

### 2.2. Nutritional Analysis

#### 2.2.1. Nutritional Profile

Moisture was determined by drying fresh biomass in a ventilated oven at 105 °C for 16 h. Freeze-dried and ground samples were analyzed for ash, by incinerating samples in a muffle furnace for 2 h at 600 °C [19], crude protein (CP), by measuring total nitrogen (N) in a CHN Elemental Analyzer (Vario EL III), and estimated by multiplying the N content by a factor of 6.25. Total lipids (TL) were determined according to a modified protocol of the Bligh and Dyer (1959) method [20], while the neutral detergent fiber (NDF), acid detergent fiber (ADF), and acid detergent lignin (ADL) were determined in agreement with the International Organization for Standardization (ISO) directives for analyzing animal feedstuffs (ISO 16472:2006, ISO 13906:2008, and ISO 13906:2008, respectively). Cellulose and hemicellulose contents were estimated by the difference between ADF and ADL or NDF and ADF, respectively.

#### 2.2.2. Mineral Content

Minerals were assessed by the microwave plasma-atomic emission spectrometer (MP-AES; Agilent 4200 MP-AES, Agilent Victoria, Australia), as described by Pereira et al. [21]. In sum, after digestion of ash samples for three times by the addition of nitric acid (67–69%) and hydrogen peroxide (30%) until complete evaporation, the dry digested samples were diluted in a known volume of 5% nitric acid solution for analysis. Results were expressed as g kg^−1^ of dry matter (DM) for macro-minerals and mg kg^−1^ DM for trace minerals.

#### 2.2.3. In Vitro Organic Matter Digestibility (IVOMD)

IVOMD was determined by the Tilley and Terry method modified by Alexander and McGowan [22]. Briefly, 500 mg of ground samples weighed to incubation flasks, in triplicate, and incubated at 39 °C for 48 h with 10 mL of rumen liquor and 40 mL of McDougall buffer. After this first incubation stage, the fermentation was stopped by adding 2.2 N HCl and then incubated with 50 mL of an acid pepsin solution for an additional 48 h. At the end, the residue obtained after filtration in a G2 crucible was dried at 105 °C, incinerated at 500 °C, weighed, and used to compute the IVOMD. The results of the method are calibrated using blank and standard feeds tests incubated simultaneously with the tested feedstuffs and the results are presented as g kg^−1^ of organic matter (OM).

### 2.3. Chemical Profiling of the Extracts

#### 2.3.1. Preparation of the Extracts

Dried samples were extracted with an 80:20 acetone:water solution (1:40, *w*/*v*) at room temperature (RT) for 16 h, under stirring. The residue was filtered (Whatman no. 4) and concentrated in a rotary evaporator under reduced pressure and temperature (approximately 40 °C). Dried extracts were dissolved in dimethyl sulfoxide (DMSO) at a concentration of 25 mg mL^−1^ and stored at −20 °C until use.

#### 2.3.2. Total Phenolic Content (TPC) 

The TPC of the extracts was estimated using the Folin-Ciocalteau (F-C) reagent [23], as described previously [24]. Briefly, 5 µL of the extracts (10 mg mL^−1^) were mixed with 100 µL of the F-C reagent (1:10 in water, *v*/*v*) in 96-well plates, and left for 10 min at RT, in the dark. Then, 100 µL of sodium carbonate (75 g L^−1^, in water) were added and the plate was incubated for 90 min, at RT, protected from light. Absorbance was measured at 725 nm in a multiplate spectrophotometer reader (Biotek Synergy 4). A calibration curve was prepared using gallic acid as a standard and TPC was expressed as gallic acid equivalents (GAE; mg GAE g extract^−1^, dry weight (DW)).

#### 2.3.3. Total Flavonoid Content (TFC) 

TFC was determined by the aluminum chloride (AlCl3) method [25]. Briefly, 50 µL of the samples at 10 mg mL^−1^ were mixed with 50 µL of 2% AlCl_3_ in a methanol and left to incubate for 10 min at RT. Absorbance was measured at 415 nm in a multiplate spectrophotometer reader. A calibration curve was prepared using quercetin as a standard and TFC was expressed as quercetin equivalents (QE; mg QE g extract^−1^, DW).

#### 2.3.4. Total Condensed Tannins Content (CTC) 

CTC was evaluated by the 4-dimethylaminocinnamaldehyde-hydrochloric acid (DMACA–HCl) colorimetric method [26] adapted to 96-well microplates [24]. In brief, 10 µL of the extracts (10 mg mL^−1^) were mixed with 200 µL of a methanol solution of DMACA (1%, *w*/*v*), and 100 µL of hydrochloric acid (37%, *v*/*v*). After a 15 min incubation period, absorbance was measured at 640 nm in a multiplate spectrophotometer reader. A calibration curve was prepared using catechin as a standard and the concentration of CT was expressed as catechin equivalents (mg CE extract^−1^, DW). 

#### 2.3.5. Phenolic Profile by High Performance Liquid Chromatography-Diode Array Detection (HPLC-DAD) 

The extracts at a concentration of 10 mg mL^−1^ in a mixture of 90% ultrapure water and 10% methanol were analyzed by HPLC-DAD (Agilent 1100 Series LC system, Germany). Analyses were performed on a MediterraneaTM sea 18 column, 15 × 0.21 cm^2^, 5 µm particle size (Teknokroma, Spain). The mobile phase consisted of a mixture of methanol (solvent A) and 2.5% acetic acid aqueous solution with the following gradient: 0–5 min: 10% A, 5–10 min: 10–30% A, 10–40 min: 30–90% A, 40–45 min: 90% A, 45–55 min: 90–10% A, and 55–60 min: 10% A, using a flow of 0.35 mL min^−1^. The injection volume was 20 μL with a draw speed of 200 μL/min. The detector was set at 210, 280 (used for quantification), 320, and 350 nm. For identification, the retention parameters of each assay were compared with the standard controls and the peak purity with the UV-visible spectral reference data. The levels of the different compounds were interpolated from calibration standard curves. Commercial standards of naringenin-7-glucoside, luteolin-7-o-glucoside, flavone, rutin, quercetin, catechin hydrate, epigallocatechin gallate, epicatechin, gallic acid, gentisic acid, p-hidroxybenzoic acid, vanillic acid, syringic acid, salicylic acid, ellagic acid, cafeic acid, coumaric acid, ferulic acid, rosmarinic acid, and chlorogenic acid were prepared in methanol (1 g L^−1^) and diluted with ultrapure water in the desired concentration.

### 2.4. Bioactive Properties

#### 2.4.1. In Vitro Antioxidant Properties 

In all the assays, the extracts were tested in serial diluted concentrations (10, 5, 2.5, 1.25, 0.625, 0.3125, 0.156, 0.078 mg mL^−1^), in order to enable the computing and calculation of the half maximal inhibitory concentration (IC_50_ value). Absorbances were measured in a multiplate spectrophotometer reader (Biotek Synergy 4). Except for the ferric reduction antioxidant power (FRAP) assay, where results were calculated in relation to the positive control, results were expressed as a percentage of inhibition in relation to the negative control (DMSO) and as IC_50_ values (mg mL^−1^), whenever possible.

##### Radical Scavenging Activity (RSA) on DPPH• Free Radical

The RSA on DPPH• free radical was determined as described elsewhere [24]. Briefly, 22 µL of the extracts were mixed with 200 µL of a methanol DPPH solution (120 µM) and left to incubate at RT in the dark. After 30 min, absorbance was measured at 517 nm. Butylated hydroxytoluene (BHT; 1 mg mL^−1^) was used as the positive control.

##### RSA on ABTS•+ Free Radical

The RSA on ABTS radical was determined as described previously [24]. A stock solution of ABTS• + (7.4 mM) was prepared by mixing ABTS with potassium persulfate (2.6 mM, in water) for 16 h in the dark, at RT. For the assay, 10 µL of the extracts, were added to 96-well plates, mixed with 190 µL of the ABTS• + solution and incubated at RT, for 6 min, in the dark. Absorbance was measured at 734 nm and BHT (1 mg mL^−1^) was used as the positive control.

##### RSA on Superoxide Anion (O_2_^−^•)

The RSA towards O_2_^−^• was evaluated according to the method described before [27]. In brief, 100 µL of the extracts were mixed with 50 µL of Tris–HCl buffer (16 mM; pH 8.0), 50 µL of nitroblue tetrazolium (0.3 mM in Tri-HCl buffer), 50 µL of nicotinamide adenine dinucleotide solution (0.936 mM in a solution of sodium hydroxide 5 mM), and 50 µL of phenazine methosulfate (0.12 mM in ultrapure water), were left to incubate for 5 min at RT. Absorbance was measured at 560 nm and ascorbic acid (1 mg mL^−1^) was used as the positive control.

##### Metal Chelating Activity on Copper (CCA) and Iron (ICA)

CCA and ICA were assayed as described elsewhere [24,28]. For CCA, the extracts (30 µL) were mixed with 200 µL of 50 mM Na acetate buffer (pH 6), 6 µL of pyrocatechol violet (4 mM) in the buffer, and 100 µL of copper sulfate pentahydrate (CuSO4.5H20; 50 µg mL^−1^, in distilled water). Absorbance was measured at 632 nm. For ICA, extracts (30 µL; 10 mg mL^−1^) were mixed with 200 µL of distilled water and 30 µL of FeCl_2_ solution (0.1 mg mL^−1^ in distilled water) and left to incubate for 30 min, before adding 12.5 µL of ferrozine (40 mM in distilled water). Absorbance was measured at 562 nm and ethylenediamine tetraacetic acid (EDTA; 1 mg mL^−1^) was used as the positive control.

##### Ferric Reducing Antioxidant Power (FRAP)

FRAP was determined as described by Rodrigues et al. [24]. Samples (50 µL) were mixed with distilled water (50 µL) and potassium ferricyanide (1% in water; 50 µL) and incubated for 20 min at 50 °C. Then, 50 µL of trichloroacetic acid (10% in water, *w*/*v*) and the ferric chloride solution (0.1% in water, *w*/*v*) were added. Absorbance was measured at 700 nm in a multiplate reader and results were expressed as a percentage in relation to the positive control (ascorbic acid, 1 mg mL^−1^), and as IC_50_ values (mg mL^−1^). 

### 2.5. In Vitro Anti-Inflammatory Properties

#### 2.5.1. Cell Viability 

The murine leukemic monocyte-macrophage cell line (RAW264.7) was provided by the Faculty of Pharmacy and Centre for Neurosciences and Cell Biology (University of Coimbra, Portugal). Cells were maintained in a RPMI culture medium supplemented with 10% heat-inactivated FBS, 1% L-glutamine (2 mM), and 1% penicillin (50 UmL^−1^)/streptomycin (50 µg mL^−1^) at 37 °C in humidified atmosphere with 5% CO_2_. Exponentially growing cells were plated in 96-well tissue plates at a concentration of 1 × 104 cells/well and incubated for 24 h to allow macrophages adhesion. Extracts were then applied at 100 µg mL^−1^ and the plate was incubated for 24 h at 37 °C 5% CO_2_. Control cells were treated with DMSO at the highest concentration used in test wells (0.5%). Cell viability was determined using the MTT reagent [29]. In brief, 2 h prior to the end of the incubation period, 20 µL of MTT (5 mg mL^−1^ in PBS) were added to each well, followed by 150 µL of DMSO, to dissolve the formazan crystals. Absorbance was measured at 590 nm in a multiplate reader. Results are expressed as a percentage of cell viability relative to a control containing DMSO (0.5% *v/v*).

#### 2.5.2. In Vitro Anti-Inflammatory Properties 

The nitric oxide (NO) production by lipopolysaccharide (LPS)-stimulated RAW 264.7 macrophages was evaluated as described by Rodrigues et al. [30]. Cells were plated at 2.5 × 105 cells/mL in 96-well tissue plates and allowed to adhere for 24 h. Extracts were then applied at 100 µg mL^−1^, in a serum- and phenol-free culture medium, containing LPS (100 ng mL^−1^), and plates were incubated for 24 h. A calibration curve was prepared using sodium nitrite as a standard and NO production was assessed using the Griess method [31]. Briefly, 100 µL of samples supernatant were mixed with 100 µL of the Griess reagent (1% (*w/v*) sulphanilamide + 0.1% of NED and 2.5% (*v/v*) phosphoric acid) and left to incubate for 20 min at RT in the dark. Results were expressed as a percentage (%) of NO production, in comparison to the non-treated LPS-stimulated control cells (0.5%, *v/v*). L-NG-Nitroarginine methyl ester (L-NAME) is a nitric oxide synthase inhibitor and was used as the positive control.

### 2.6. Statistical Analysis

All the experiments were performed, at least, in duplicate. Data concerning the nutritional profile, IVOMD, and mineral content of the biomass are expressed as mean. The phenolic content and nitric oxide production results are expressed as mean ± standard error of the mean (SEM), while results on the antioxidant properties are expressed as the concentration that results in a 50% inhibition (IC_50_). IC_50_ values were obtained by sigmoidal fitting of the data, using the GraphPad Prism Software v.5.0. Seasonal effects were analyzed using the IBM SPSS Statistics v. 20.0 software, by analysis of variance (ANOVA) and the significance between means was explored using the post-hoc Tukey HSD test, at a significance value of 0.05.

## 3. Results and Discussion

### 3.1. Nutritional Profile 

Table 1 summarizes the results on the seasonal variation of the nutritional profile of sawgrass biomass. As expected, the nutritional profile of sawgrass varied among seasons. Dry matter (DM) was higher in summer (586 g kg^−1^) and autumn (560 g kg^−1^) than in spring (449 g kg^−1^) and winter (469 g kg^−1^). The ash level peaked in autumn (82 g kg^−1^ DM) and was minimum in summer (49 g kg^−1^), during the heading and seed ripe stage, similar to other grasses and sedges [32]. The ash content of sawgrass was lower than those reported for other salt-tolerant plants [5,33], such as species belonging to Chenopodiaceae, Juncaceae, Tamaricaceae, and Zygophyllaceae genera (ranging from 122–403 g kg^−1^). This is probably due to different approaches in use by grasses for osmotic adjustment to cope with salinity levels, e.g., water loss vs. ion accumulation [34].

Crude protein (CP) is often used as a major indicator of forage quality as is correlated with vegetative vigor [35]. Sawgrass aerial organs present a very low CP during all seasons (51–57 g kg^−1^ DM) except in Spring when it is slightly higher (87 g kg^−1^ DM). Its CP content is similar to that observed for other salt-tolerant species studied as potential forages for ruminants, such as *Halocnemum strobilaceum* (Pall.) M.Bieb. (67 g kg^−1^ DM), *Juncus acutus* L. (71 g kg^−1^ DM), *Salsola tetandra* Forssk. (63 g kg^−1^ DM), and *Zygophyllum album* L. (78 g kg^−1^ DM) [5] but lower than *Cynodon dactylon* (Bermuda grass; 98 g kg^−1^) [36], *Atriplex amnicola* (161 g kg^−1^ DM) [37] or *A. halimus* (167 g kg^−1^ DM) [38]. Still, it is a low value when compared with Mediterranean rainfed pastures (73 up to 500 g kg^−1^ DM) in which the lowest CP observed in summer are comparable to the highest CP of sawgrass observed in Spring [39]. In agreement, sawgrass CP contents are similar to those observed for some sedge species with a low forage potential such as *Carex vulinoidea* (58–110 g kg^−1^ DM) and *C. aenea* (59–75 g kg^−1^ DM) [40].

High levels of fiber, including NDF (596–690 g kg^−1^), ADF (330–418 g kg^−1^), and ADL (5–24 g kg^−1^) were observed for all seasons, with a peak in summer, probably as a result of the reproductive stage. Catling et al. (1994) observed a significant increase throughout summer in the ADF contents of *Carex* sedge species, coupled with a decline in digestibility and CP [40]. However, these changes can be a result of a combination of different factors, such as plant maturity, meteorological changes or harvest collection [40]. Aerial parts are also characterized by quite low IVOMD, which varied from 171 g kg^−1^ OM in summer to 317 g kg^−1^ OM in spring. Fiber is often the bulk of ruminant’s diets and its digestibility is determinant of the forage’s quality. The concentration and type of structural polysaccharides, as cellulose and hemicellulose, and the degree of its lignification determines its digestibility and hence its usefulness of the forage for supporting ruminant production [41]. In this work, the very low IVOMD is correlated to the high NDF (Pearson’s correlation, r = −0.958; *p* < 0.05) and ADF (r = −0.994; *p* < 0.05) results. A negative correlation between organic matter digestibility with cell wall fractions, particularly NDF, is well recognized [42]. The fiber and digestibility values of sawgrass are similar to that of other *Carex* sedge species (ADF, 276–409 g kg^−1^; digestibility, 203–337 g kg^−1^ OM) [40]. Still, the IVOMD result obtained in summer (172 g kg^−1^ OM) emphasizes its poor digestibility, since it is even lower than the 77 *Carex* species studied [40]. On the other hand, a high content of secondary metabolites, such as condensed tannins, might also limit digestibility. Despite the fact that high tannin contents (>5–6%) may impair feed intake, animal productivity, and digestion, moderate amounts (2–5%) exert beneficial effects such as improving protein and ruminal metabolism and reducing methane emissions, consequently increasing the overall nutritive value [43,44]. The CTC in *C. mariscus* biomass was estimated around 2–4% DM (data not shown), and therefore, its impact on digestibility is expected to be minor. Nevertheless, in addition to the concentration, the chemical structure of tannins present as well as the diet composition will also be a determinant of its benefits in ruminant nutrition and health [45].

The mineral composition of sawgrass is presented in Table 1. All macro- and trace minerals were within the maximum tolerable levels, reported by the National Research Council, for ruminants [46]. Minerals have a key role in structural, physiological, and regulatory processes, and thus mineral deficiency may have a significant negative impact in animal health and performance [47]. The seasonal distribution of macro- and trace mineral contents had dispersed patterns throughout the seasons: high amounts of Na and Ca were noted in spring; K and Fe were higher in spring/summer; Zn only decreased in spring; Cr was particularly higher in summer; Mn increased in spring/winter; while Mg was stable throughout the seasons. The salt (sodium chloride) content is one of the major drawbacks of using salt-tolerant plants as forages for livestock [48]. In the case of sawgrass, the Na concentration increased in spring (8.1 g kg^−1^ DM) and was lowest in summer (1.4 g kg^−1^ DM). The negative effects of high Na dietary levels on feed intake and animal performance are negligible if water is freely available. Moreover, forages are often deficitary in Na and salt supplementation of ruminants is usually needed [49]. Potassium (K) values peaked in spring (4.4 g kg^−1^ DM) and decreased in the other seasons. The magnesium (Mg) content did not differ among seasons (*p* > 0.05) but the calcium (Ca) levels were lower in summer (1.6 g kg^−1^ DM) and peaked in spring (6.9 g kg^−1^ DM). Having in mind the macro-mineral concentration requirements for ruminants [50], sawgrass aerial organs are able to supply dietary levels of Ca and Na, while low levels of Mg and K are observed. Regarding trace minerals, sawgrass biomass has adequate levels of iron (Fe), the main component of hemoglobin and myoglobin, particularly in summer and spring periods (186–214 mg kg^−1^ DM). Along with other elements, zinc (Zn), manganese (Mn), and copper (Cu) participate in antioxidant defense mechanisms and thus, a balanced supply of these elements is important during ruminant production [51]. Mn levels were higher in summer and winter seasons (40.7–49.0 mg kg^−1^ DM), while the Zn and Cu contents increased in winter (Zn, 24.7 mg kg^−1^ DM; Cu, 9.6 g kg^−1^) and dropped in spring (Zn, 15.2 mg kg^−1^ DM; Cu, 3.9 g kg^−1^). Although Zn levels are low, in comparison to other highly salt-tolerant plants [51], Mn and Cu results present similar contents. Overall, Fe and Mn concentrations of sawgrass are within the threshold of dietary levels in forages for ruminants [52]. It is important to take into consideration that the concentration of these elements is dependent on soil and plant related factors [47,53,54], and consequently may vary greatly.

Altogether, the results on the nutritional profile and mineral content of sawgrass biomass indicate that it is a low-quality roughage with limited interest as a nutrient supplier for ruminants. This is in agreement with other sedge species (Cyperaceae) which have been pointed out as used for animal forage and grazed by ruminants, but the majority are reportedly of poor-quality [40,55].

A comparison of our results with others is hampered by the lack of published data concerning *C. mariscus*, particularly its nutritional value and mineral content. Only one study analyzed the nutlets, rhizomes, and culms of *C. mariscus* collected in South Africa [56]. Sievers (2015) showed that the nutlets are not a rich source of protein (30 g kg^−1^) or fat (3 g kg^−1^) but have a high content of carbohydrates (490g kg^−1^ DM), however digestible and undigestible fiber contents were not addressed [56].

### 3.2. Phenolic Content of the Extracts

Phenolic compounds are a major group of plant secondary metabolites with a panoply of well documented bioactive properties including antioxidant, anti-inflammatory, and antimicrobial. The use of phenolics or phenolic-rich plants (particularly in tannins and flavonoids) as natural alternatives to improve animal nutrition, health, and productivity has gained the interest of the scientific community in the last decades [11,12,13,14]. Therefore, the identification of species which are rich sources of phenolic compounds and its characterization meets the current research agenda on ruminant production.

**Figure 1:**
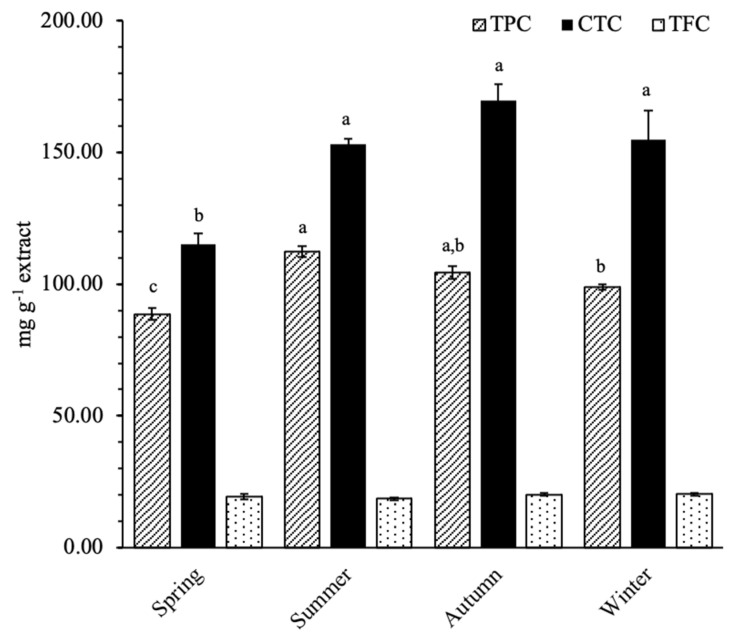
Seasonal variation of the total phenolic levels of sawgrass (*C. mariscus*) aqueous acetone extracts. TPC: Total phenolic content, expressed as mg gallic acid equivalents g^−1^ extract (mg GAE g^−1^, DW); CTC: Condensed tannins content, expressed as mg catechin equivalents g^−1^ extract (mg CE g^−1^, DW); TFC: Total flavonoid content, expressed as mg quercetin equivalents g^−1^ extract (mg QE g^−1^, DW). Values are expressed as mean with standard deviation of the mean represented (n = 12). Different letters superscript represent significant differences among seasons, for each assay (*p* < 0.05; Tukey HSD).

Figure 1 represents the seasonal variations of the total phenolic content of sawgrass extracts, determined by spectrophotometric methods. A high TPC was noted for all samples (>20 mg g^−1^ extract) [44] and it was significantly increased in summer and autumn samples (112.32–104.3 mg GAE g^−1^ extract) in contrast to spring (88.6 mg GAE g^−1^ extract). Similarly, CTC was lower in spring (115.1 mg CE g^−1^ extract) but remained stable in the other seasons (153.1–169.6 mg CE g^−1^ extract). No significant differences were recorded for TFC, throughout the seasons (18.5–20.2 mg QE g^−1^ extract).

Seasonal variations on the TPC and CT contents of *C. mariscus* were expected, having in mind that the phenolic content of plant tissues is influenced by the climate, geography, type of organ, nutrient-related stress, and salinity, as well as laboratory drying and extraction methodologies [54,55,56,57]. Thus, the increased levels noted for summer and autumn periods could be a result of the plant response to counteract the environmental challenges during these seasons, e.g., drought and high UV exposure. Moreover, phenolics biosynthesis is also known to be enhanced during metal stress, aiming for the protection of the plant from oxidative stress [58], and, therefore, the levels of Zn, Cu, and Cr during summer and/or autumn seasons may have contributed to this augmented phenolic concentration. On the other hand, during these periods, inflorescences were collected along with leaves, and thus, the possibility of organ-related variations should not be excluded.

Lopes et al. presented higher TPC (254 mg GAE g^−1^ extract) but lower TFC (13.8 mg RE eq. g^−1^ extract) and CTC values (38.7 mg CE g^−1^ extract) [9]. These differences may be explained by the different extraction methodologies applied by those authors, i.e., 70% acetone extracts, but also environmental and organ-related factors, i.e., leaves collected in June 2013 whilst aerial parts collected amongst different seasons. The high CTC content of sawgrass extracts is particularly interesting: despite the fact that it might contribute to its very low digestibility it also holds potential applications. Tannins have been perceived, for a long time, as anti-nutritional metabolites, mainly due to its negative effects on animal feed intake and nutrient digestibility [59]. However, there is a body of evidence showing that tannins, in the right dosage, also have important positive effects on animals, including the decrease of methane production, control of gastrointestinal parasites, preventing bloat, and improving meat fatty acid composition [6,11,15,45,59].

**Figure 2:**
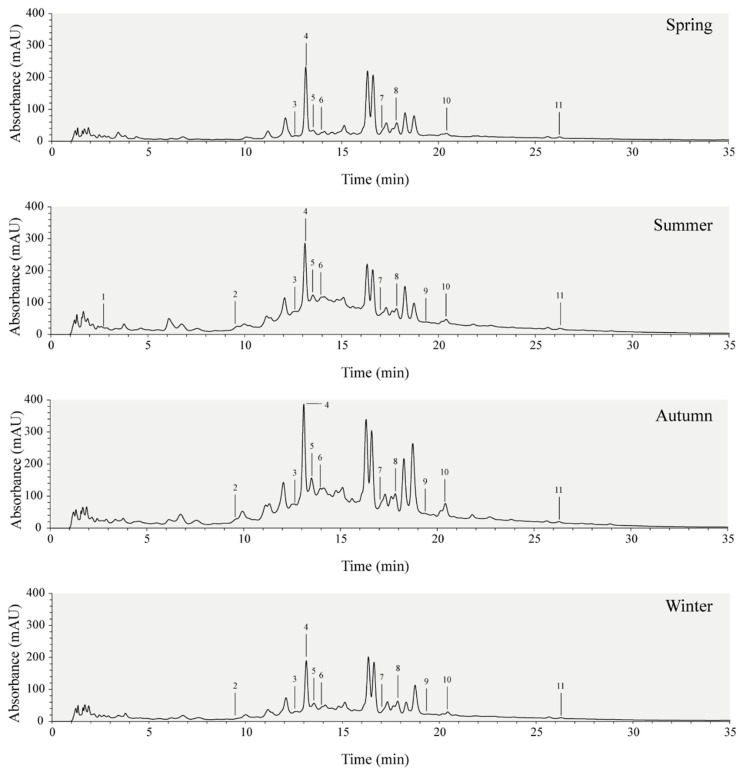
HPLC-DAD chromatograms of sawgrass (*C. mariscus*) acetone water extracts throughout the seasons.

Aiming to identify and quantify individual phenolics, samples were subjected to HPLC-DAD analysis, as summarized in Table 2 and chromatograms represented in Figure 2. In agreement with our previous spectrophotometric results, the summer and autumn samples exhibit increased levels of phenolics (Figure 2). Eleven compounds were identified and quantified, specifically five flavonoids (catechin, epicatechin, naringenin-7-glucoside, luteolin-7-*O*-glucoside, and quercetin), and six phenolic acids, namely gallic, caffeic, chlorogenic, syringic, ferulic, and salicylic acids (Table 2). These compounds are, to the best of our knowledge, herein firstly identified and quantified in sawgrass. 

Phenolic compounds play a major role in plant defense mechanisms against biotic and abiotic stressors and thereof, it was anticipated that their concentration would vary accordingly.

Chlorogenic acid (CGA) was the most abundant compound in all samples with concentrations ranging from 2.29 up to 4.45 mg g^−1^ extract, marking the lowest value in winter. CGA is a strong antioxidant compound produced under environmental stresses such as boron and nitrogen deficits and intense UV radiation [60,61,62]. In agreement, Meot-Duros and Magné (2009), found that *Crithmum maritimum* L. plants living in sand hills, a stressful habitat, had increased amounts of CGA than those living below cliffs [63]. In this study, the variability of CGA in sawgrass extracts may be linked to the increased oxidative stresses developed in response to the higher temperatures, UV radiation, and lower precipitation values in summer and autumn. 

Other phenolic acids quantified are salicylic (1.64–2.92 mg g^−1^ extract), ferulic acid (1.38–2.40 mg g^−1^ extract), and syringic acid (0.19–0.73 mg g^−1^), all present in higher concentrations in the autumn sample. Similarly, to CGA, salicylic acid, an important regulator of plant growth, is also involved in the induction of defense responses and tolerance to drought [64], heavy metal tolerance [65], salinity [66], and heat [67]. On the other hand, syringic and ferulic acids are important contributors to the structure integrity of lignin, providing overall rigidity and strength to the plant cell wall [68,69]. Thus, the accumulation of these compounds during the dry seasons can also be linked to an increase in cell wall lignification, in response to abiotic stressful conditions [70]. In addition to their physiological role, these phenolic acids have documented bioactivities of pharmacological importance such as antioxidant, anti-inflammatory, hepatoprotective, cardioprotective, and antimicrobial [69,71,72].

Besides phenolic acids, plants inhabiting the harsh Mediterranean environmental settings, particularly excessive sunlight and drought/salinity, also accumulate higher amounts of UV-absorbing and antioxidant flavonoids and its glycosides and other polyphenols, which play a role in the photoprotection of the plant [73]. In accordance, in this work, the flavonoids naringenin-7-glucoside, luteolin-7-*O*-glucoside, catechin, epicatechin, and quercetin were mostly increased in seasons with higher light irradiance and UV exposure. Luteolin-7-*O*-glucoside concentration was significantly enhanced in bell pepper leaves after UV-B exposure, probably due to its high antioxidant capacity needed to quench the reactive oxygen species (ROS) produced [74]. In addition to antioxidant and anti-inflammatory activities [75], luteolin-7-*O*-glucoside also exhibits in vitro methane and ammonia mitigating properties [76], and quercetin, luteolin, and naringenin display in vitro anthelmintic properties against gastrointestinal parasites of small ruminants [77].

In total, a higher amount of identified compounds was quantified in autumn (14.55 mg g^−1^ extract) compared to the other seasons (8.43–9.22 mg g^−1^ extract). Nonetheless, due to the complexity of the extract mixtures, it is worth mentioning that a large number of compounds remain to be identified, including two major metabolites (e.g., retention time between 16–17 min; Figure 2), which is already being pursued by additional hyphenated methods.

### 3.3. Bioactive Properties 

As in other animals, the oxidative stress in ruminants is mainly associated with metabolic and inflammatory disorders, environmental factors (e.g., heat stress), and dietary imbalances [78], and can significantly impair important physiological and immunological functionalities, compromising animal welfare and performance [79,80]. Former reviews point out to the beneficial effects of using antioxidant supplementation, particularly for improving the animal general antioxidant status in reproductive-related events (e.g., lactation, fertility) [80,81], inflammatory processes (e.g., mastitis, parasitic infections) [82,83], and on enhancing the quality of derived products (meat, milk) [79]. Additionally, there is an increasing interest in using bioactive plants, rich in antioxidants, such as phenolics, for the latter purpose [10,78,84].

**Figure 3:**
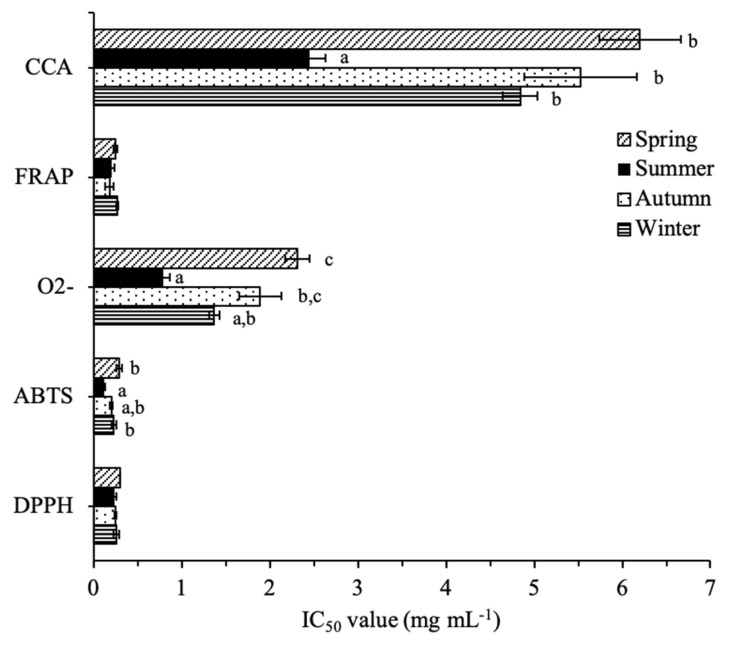
Seasonal variation of the antioxidant capacity of sawgrass (*C. mariscus*) extracts. Values are expressed as IC_50_ with the standard deviation represented (n = 3). Different letters superscript correspond to significant differences between seasons, for each assay (*p* < 0.05; Tukey HSD). CCA: Copper chelating activity; FRAP: Ferric reduction antioxidant power.

Seasonal variations on the antioxidant capacity of the extracts are represented in Figure 3. All the samples were effective on scavenging DPPH^•^ and ABTS^•+^ radicals (IC_50_ < 0.30 mg mL^−1^). While no statistical differences for the activity towards DPPH^•^ were observed, the ability to scavenge ABTS^•+^ was higher in summer (IC_50_ = 0.12 mg mL^−1^), with an IC_50_ value comparable to the positive control, BHT (IC_50_ = 0.10 mg mL^−1^). Similarly, the summer sample was significantly more efficient on scavenging the O_2_^−•^ radical (IC_50_ = 0.79 mg mL^−1^) and on chelating copper (CCA; IC_50_ = 2.45 mg mL^−1^). In contrast, the FRAP activity remained unchanged throughout the seasons (IC_50_ = 0.18–0.27 mg mL^−1^) and none of the samples was able to chelate iron at the maximum concentration tested (10 mg mL^−1^). The variability amongst the antioxidant activity results obtained, emphasize the importance of using different in vitro methodological approaches [85] due to the dissimilarities among the test systems in this study. Lopes et al. reported an IC_50_ value of 0.23 mg mL^−1^ towards the DPPH^•^ radical, for a 70% acetone extract of leaves of the same species, similar to those obtained in this study (0.24–0.30 mg mL^−1^) [9]. However, the IC_50_ value presented against the ABTS^•+^ radical (0.32 mg mL^−1^) was slightly higher in comparison to our results, especially with the summer sample (0.12 mg mL^−1^). As previously mentioned, besides environmental and methodological variations, the collection of inflorescences during the summer period could have contributed to this difference. Having in mind that a correlation between TPC and antioxidant activity has been previously reported in the literature [85,86], the generally greater antioxidant activity noted in summer can most probably be attributed to the augmented phenolic content noted in this period. In fact, Meot-Duros and Magné (2009) found a linear correlation not only between DPPH and ABTS anti-radical activity of *C. maritimum* leaves and total phenolics, but particularly with CGA [63]. Furthermore, anti-radical and total phenols were increased in summer in detriment to the winter samples [63]. Despite the fact that several phenolics identified in this study have described the antioxidant activity in literature, CGA was present in higher amounts, and thus is most probably significantly contributing to the total antioxidant capacity observed, particularly in summer. Nevertheless, one should not reject the possibility of other phytochemicals to be present that may also contribute to this activity and/or of synergistic effects between these metabolites.

**Figure 4:**
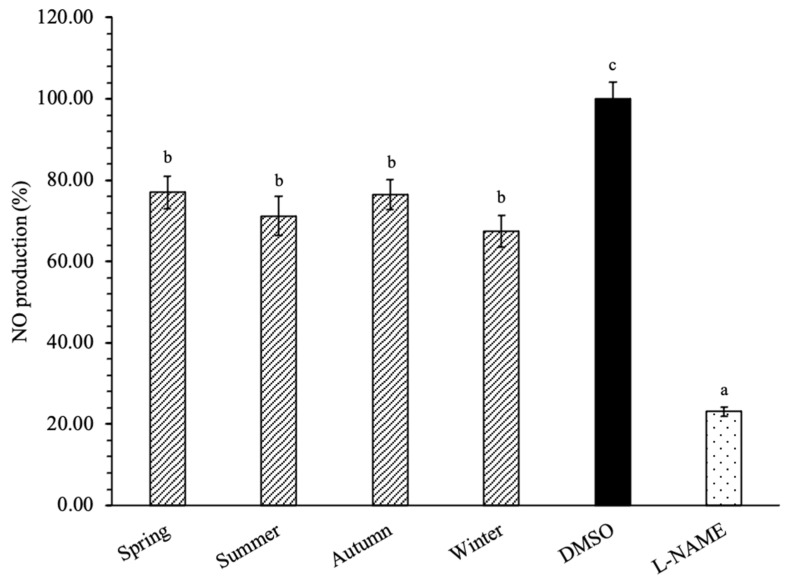
Season effects on nitric oxide (NO) production by lipopolysaccharide (LPS)-stimulated RAW 264.7 macrophages (%) of sawgrass (*C. mariscus*) extracts, applied at 100 µg mL^−1^ (n = 12). L-NAME (L-N^G^-Nitro arginine methyl ester) was used as the positive control (100 µg mL^−1^). Different letters correspond to significant differences between the samples (*p* < 0.05).

Biswas (2016) highlights that oxidative stress inflammation are closely related and associated events and, therefore, the identification of bioactive agents targeting both processes is relevant [87]. NO plays a pivotal role in the mediation of immune response and inflammation, and high levels are detected in several ruminant inflammatory processes, such as mastitis and reproductive disorders, in which oxidative stress is also involved [82]. In this work, and despite the fact that no statistical differences were recorded amongst the seasons, the extracts were able to reduce NO production by 30%, in comparison to the control cells (Figure 4). Among the phenolic compounds previously identified in sawgrass extracts, the majority are well recognized for its anti-inflammatory effects, such as syringic acid [69], ferulic acid [72], and luteolin-7-*O*-glucoside [75]. The chlorogenic acid inhibits NO production, COX-2 and iNOS expression, pro-inflammatory cytokines production, and nuclear translocation of NF-κB, with pronounced effects at 20 µM [88]. Additionally, anti-inflammatory phenolic compounds may act together: For example, quercetin and catechin exhibit synergistic anti-inflammatory effects in LPS-stimulated macrophage RAW 264.7 cells by decreasing NO, TNF-α, IL-1β, iNOS, COX-2, inhibiting NF-κB, as well as the activation of LR4–MyD88-mediated NF-κB and the expression of mitogen-activated protein kinases [89].

Although in this study the extracts were tested at a maximum concentration of 100 µg mL^−1^, further investigations including higher concentrations may unveil increased anti-inflammatory properties, as long as no cytotoxic effects remain.

## 4. Conclusions

Sawgrass biomass has a chemical composition and mineral content similar to that of other roughage resources characterized by low crude protein, high indigestible fiber contents, very low in vitro digestibility, and therefore has limited interest as a nutrient supplier for ruminants. On the other hand, acetone water extracts from its biomass confirmed this species as a rich source of antioxidant and anti-inflammatory phenolic compounds particularly in summer and autumn samples, that can be valuable assets in integrated control strategies on the management of oxidative stress and inflammatory related disorders affecting ruminant’s health, productivity, and performance. Further studies should be pursued to deepen the knowledge on its potential veterinary phytotherapeutic applications. 

## Figures and Tables

**Table 1:** Seasonal effects on the nutritional value, in vitro digestibility, and mineral content of aerial parts of sawgrass (*C. mariscus*) biomass.

	Spring	Summer	Autumn	Winter
**Nutritional profile** (g kg^−1^ DM)				
DM	449	586	559	469
Ash	71.7	49.4	82.9	75.1
CP	87.3	51.8	54.5	57.8
TL	53.3	48.9	52.9	51.1
NDF	596	690	628	613
ADF	330	418	380	363
ADL	8	24	8	5
Cellulose	322	393	372	358
Hemicellulose	266	272	248	250
**IVOMD** (g kg^−1^ OM)	317	172	243	255
*Mineral content*				
**Macro-minerals** (g kg^−1^ DM)				
Ca	6.9	1.6	3.8	3.8
K	4.4	3.9	2.3	2.4
Mg	0.8	0.7	0.7	0.7
Na	8.1	1.43	5.7	4.9
**Trace minerals** (mg kg^−1^ DM)				
Fe	186	214	42.8	32.3
Zn	15.2	21.1	20.4	24.7
Cu	3.9	7.7	5.9	9.6
Mn	40.7	20.0	29.1	49.0
Cr	4.3	13.0	1.7	2.3

DM: Dry matter; OM: Organic matter; CP: Crude protein; TL: Total lipids; NDF: Neutral detergent fiber; ADF: Acid detergent fiber; ADL: Acid detergent lignin; IVOMD: In vitro organic matter digestibility; Ca: Calcium; K: Potassium; Mg: Magnesium; Na: Sodium; Fe: Iron; Zn: Zinc; Mn: Manganese; Cr: Chromium.

**Table 2:** HPLC-diode array detection (HPLC-DAD) identification and quantification (mg g^−1^ extract) of the phenolic compounds of sawgrass (*C. mariscus*) extracts, for the different seasons. Peaks are numbered according to its retention time, in ascending order.

Phenolic Group	RT (min)	Compound	Peak	Spring	Summer	Autumn	Winter
*Flavonoids*							
Flavanone	19.3	Naringenin-7-*O*-glucoside	**9**	<0.01	0.01	0.03	0.01
Flavone	20.4	Luteolin-7-*O*-glucoside	**10**	0.16	0.46	0.98	0.24
Flavanols	26.2	Quercetin	**11**	0.03	0.03	0.03	0.01
	9.5	Catechin hydrate	**2**	-	0.77	0.76	0.10
	13.9	Epicatechin	**6**	0.42	0.88	1.49	1.06
*Phenolic acids*							
Hydroxybenzoic acids	2.6	Gallic acid	**1**	<0.01	0.01	<0.01	<0.01
	8.5	p-Hidroxybenzoic acid	-	-	<0.01	<0.01	-
	13.5	Syringic Acid	**5**	0.19	0.35	0.73	0.35
	17.8	Salicylic Acid	**8**	2.11	1.64	2.92	2.09
	22.3	Ellagic Acid	-	<0.01	-	-	-
Hydroxycinnamic acid	12.6	Cafeic Acid	**3**	0.67	0.73	0.76	0.70
	15.8	Coumaric Acid	-	<0.01	<0.01	<0.01	<0.01
	16.9	Ferulic Acid	**7**	1.88	1.38	2.40	1.58
	13.1	Chlorogenic Acid	**4**	3.03	2.96	4.45	2.29
**Other**	10.3	4-Hydroxybenzaldehyde	-	<0.01	<0.01	<0.01	-
		∑ Phenolics		**8.49**	**9.22**	**14.55**	**8.43**

RT: Retention time; -: Not detected.
